# A Rapid and Efficient Immunoenzymatic Assay to Detect Receptor Protein Interactions: G Protein-Coupled Receptors

**DOI:** 10.3390/ijms15046252

**Published:** 2014-04-11

**Authors:** Elisa Zappelli, Simona Daniele, Maria P. Abbracchio, Claudia Martini, Maria Letizia Trincavelli

**Affiliations:** 1 Department of Pharmacy, University of Pisa, 56126 Pisa, Italy; E-Mails: elisa.zappelli@for.unipi.it (E.Z.); simona.daniele@for.unipi.it (S.D.); maria.trincavelli@farm.unipi.it (M.L.T.); 2 Department of Pharmacological and Biomolecular Sciences, University of Milan, 20133 Milan, Italy; E-Mail: mariapia.abbracchio@unimi.it

**Keywords:** immunoenzymatic assay, G protein coupled-receptors, protein-protein interactions

## Abstract

G protein-coupled receptors (GPCRs) represent one of the largest families of cell surface receptors, and are the target of at least one-third of the current therapeutic drugs on the market. Along their life cycle, GPCRs are accompanied by a range of specialized GPCR-interacting proteins (GIPs), which take part in receptor proper folding, targeting to the appropriate subcellular compartments and in receptor signaling tasks, and also in receptor regulation processes, such as desensitization and internalization. The direction of protein-protein interactions and multi-protein complexes formation is crucial in understanding protein function and their implication in pathological events. Although several methods have been already developed to assay protein complexes, some of them are quite laborious, expensive, and, more important, they do not generate fully quantitative results. Herein, we show a rapid immunoenzymatic assay to quantify GPCR interactionswith its signaling proteins. The recently de-orphanized GPCR, GPR17, was chosen as a GPCR prototype to optimize the assay. In a GPR17 transfected cell line and primary oligodendrocyte precursor cells, GPR17 interaction with proteins involved in the typical GPCR regulation, such as desensitization and internalization machinery, was investigated. The obtained results were validated by co-immunoprecipitation experiments, confirming this new method as a rapid and quantitative assay to study protein-protein interactions.

## 1. Introduction

G-Protein-coupled receptors (GPCRs) are members of a family of transmembrane proteins that mediate the transmission of a wide range of extracellular signals to the intracellular space, and initiate signalling mechanisms that regulate multiple cellular processes [1]. Their importance in health is underscored also by the fact that at least one-third of the currently marketed pharmaceutical agents target GPCRs [2].

Besides receptor homo-dimers and hetero-dimers, GPCRs can interact with a wide range of either soluble or transmembrane proteins, generally called GIPs (GPCR-interacting proteins) [3]. These proteins are involved in a variety of receptor processes, such as targeting of GPCRs to specific subcellular compartments, controlling their trafficking to and from the plasma membrane, as well as fine-tuning their signaling properties. GPCRs translate extracellular stimuli into intracellular signals, and the intensity and duration of these are determined by complex regulation mechanisms [3], which imply the physical interaction of GPCRs with regulatory proteins such as G protein-coupled receptor kinases (GRKs) and β-arrestins [4–6].

At the light of these evidences, it is clear that detecting protein-protein interactions and multi-protein complexes formation is crucial to understand protein function and cell biology. A large number of methods have been developed over the years to study protein-protein interactions. For example, the real-time formation of dynamic GPCR-protein complexes in living cells has been monitored by methodologies, such as fluorescence resonance energy transfer (FRET) and bioluminescence resonance energy transfer (BRET) [7].

Technology development has, in many cases, been fueled by the extraordinary advances in Mass Spectrometry (MS) [8]. Recently, proteomic approaches combining affinity chromatography and/or co-immunoprecipitation and MS have proven their efficacy in characterizing multi-protein complexes. Somewhat surprisingly, only a small number of the interactions are supported by more than one method [9]. Estimates of 40%–80% false negatives and 30%–60% false positives have been assigned to high-throughput studies that have used two-hybrid techniques, affinity based techniques or computational approaches [9–11]. Moreover, some of these methods are quite laborious, expensive, and do not generate fully quantitative results.

Herein, we describe the development of a simply immunoenzymatic assay to detect GPCR interactions with its regulatory proteins. This new method allows to skip the time-consuming steps of co-immunoprecipitation assay, and to obtain a quantitative measure of the protein-protein interaction.

In order to optimize and validate the ELISA assay, the recently deorphanized GPCR, GPR17 [12], was chosen a GPCR prototype. Both a transfected cell line and primary oligodendrocyte precursor cells (OPCs) which natively express the receptor at high levels [13], were used: in these cell models, we investigated GPR17 interactions with proteins involved in the typical GPCR desensitization and internalization machinery, upon receptor stimulation with its putative ligands [12,14,15].

The results obtained in the ELISA assay were confirmed by co-immunoprecipitation experiments, validating the new method as a rapid and quantitative assay to study protein-protein interactions. This ELISA assay can be applied to every GPCR, in the study of the interaction with signaling proteins, as well as with other GPCRs (heterodimers).

## 2. Results and Discussion

### 2.1. ELISA Assay Optimization

A quantitative sandwich immune-enzymatic assay technique on crude cell lysates was developed to study protein-protein interactions.

The following steps describe the general protocol used to investigate the association between two proteins (A and B). The first step consisted in wells’ pre-coating with an anti-A antibody. Then, cell lysates, containing the protein complex (A–B) of interest, were subsequently incubated on the pre-coated wells. After removing unbound protein A, each well was treated with BSA, to block non-specific sites, and then incubated with the appropriate primary antibody, directed toward protein B. Wells were washed, probed with a secondary HRP-conjugate antibody, and washed again. Blanks were obtained processing cell lysates in the absence of the primary anti-B antibody.

As final step, the HRP substrate (TMB substrate kit, Thermo Fisher Scientific Inc., Waltham, MA, USA) was added to the wells and then neutralized with HCl. The absorbance at 450 nM was measured in a colorimetric assay with an automated plate reader, allowing a quantification of the examined protein complex.

To avoid cross interactions between the different antibodies, the chosen primary antibody was always from a different species with respect to the antibody used for well pre-coating. When available, a monoclonal antibody was preferable to polyclonal antibody, since, by recognizing a single epitope, it provides higher specificity. Anyway, all candidate antibodies should be tested for their specificity. In particular, before its use in the ELISA assay, the chosen antibody should be tested in cells or tissues not expressing the target. As additional control, the antibody could be pre-absorbed with its blocking peptide, to ensure the specificity of the detected signal.

The ELISA method was used to investigate the association between GPR17 [12], chosen as a GPCR prototype, and its interacting proteins (*i.e.*, GRKs and β-arrestins). Cell lysates were obtained from 1321N1 cells, transfected with a HA Tagged form of the receptor, and from primary OPCs, which natively express GPR17 at high levels [13].

#### 2.1.1. Wells’ Coating

As a first step, preliminary experiments were performed to set-up the conditions of the wells’ coating: an anti-HA Tag antibody (#05-904, Millipore, Billerica, MA, USA) was chosen for 1321N1 HA Tag GPR17 transfected cells, while in the case of primary OPCs an antibody raised against a peptide mapping an epitope near the *N*-terminus of GPR17 (sc-74792, Santa Cruz Biotechnology, Santa Cruz, CA, USA) was used. The results revealed optimal coating by an antibody dilution in 0.05% Poly-l-Ornithine (1:500 and 1:100 for anti-HA Tag and anti-GPR17 antibody, respectively).

#### 2.1.2. Protein Dependence

**Figure 1.**
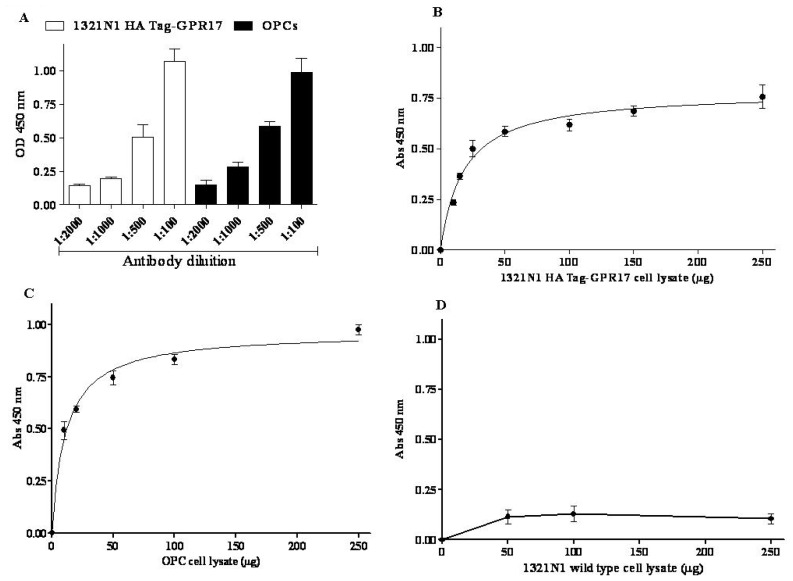
(**A**) Cell lysates (10 μg) obtained from 1321N1 HA Tag-GPR17 cells (white bars) or primary OPCs (black bars) were captured on wells pre-coated with anti-HA Tag antibody or anti-*N* terminus-GPR17 antibody, respectively. Different-fold dilutions of a full-length anti-GPR17 antibody were probed in 120 min blocking time. The secondary HRP-conjugated antibody and the TMB substrate kit allowed a colorimetric quantification of signals. Blanks were obtained processing cell lysates in the absence of the primary anti-GPR17 antibody; (**B**,**D**) Different amount of cell lysates obtained from 1321N1 HA Tag-GPR17 (**B**), or 1321N1 wild type cells (**D**) were captured on wells pre-coated with anti-HA Tag antibody. After extensive washes, levels of GPR17 were quantified using a full-length anti-GPR17 antibody, and subsequently an HRP-conjugated antibody/TMB substrate kit; (**C**) Different amounts of cell lysates obtained from primary OPCs were captured on wells pre-coated with anti-*N* terminus-GPR17 antibody. After extensive washes, levels of GPR17 were quantified using a full-length anti-GPR17 antibody, and subsequently an HRP-conjugated antibody/TMB substrate kit. Blank wells were obtained in the absence of the full length anti-GPR17 antibody. Data are expressed as specific absorbance at 450 nm and represent the mean ± SEM of at least three independent experiments.

Then, the protein dependence of the assay was revealed by adding increased aliquots of cell lysates (5 to 250 μg), obtained from 1321N1 HA Tag GPR17 transfected cells or from OPCs. The amount of receptor bound to pre-coated wells was quantified using a home-made full length anti-GPR17 polyclonal antibody [16]. Different dilutions of the primary antibody (1:100 to 1:2000) and blocking time of primary incubation (120 and 30 min) were tested. Satisfactory results were obtained with a 1:500 dilution of the primary antibody, followed by 120 min of blocking time (Figure 1A).

Specific absorbance values at 450 nm proportionally increased with protein concentration of 1321N1 HA Tag-GPR17 cell lysates (Figure 1B) or of OPC lysates (Figure 1C), with a trend toward hyperbole saturation starting from 50 μg of proteins. Non-specific absorbance at 450 nm (blank wells), obtained in the absence of primary anti-GPR17 antibody, remained always under about the 20% of total values.

In order to validate the experimental method, 1321N1 wild type cells, which did not express HA Tag GPR17, were used in the ELISA assay. Increasing aliquots of 1321N1 wild type cell lysates were incubated on wells pre-coated with anti-HA Tag antibody, and the assay was performed as described above. No specific absorbance at 450 nm was detected (Figure 1D), confirming the specificity of the assay.

### 2.2. Use of the ELISA Assay in the Study of GPR17 Association with GRKs

In previous studies on either recombinant [17] or native [18] GPR17, it has been demonstrated that receptor agonists induce GPR17 desensitization and internalization. Typically, GPCR desensitization process is consequent to receptor phosphorylation mediated by specific kinases, named GRKs, which bind the receptor and trigger the recruitment of β-arrestins. On this basis, we investigated GPR17 association with GRKs upon receptor stimulation with its endogenous ligands, UDP-glucose or LTD_4_. 1321N1 HA Tag transfected cells and OPCs were treated with UDP-glucose or LTD_4_, and cells were collected and lysed. Cell lysates were then incubated on pre-coated wells; after three quick washes to remove unbound GPR17, the presence of GPR17/GRK complexes was evaluated using specific primary anti-GRK2 or anti-GRK5 antibodies, and monitoring the increase of specific absorbance at 450 nm after the addition of the appropriate HRP-conjugate secondary antibody/TMB substrate.

**Figure 2.**
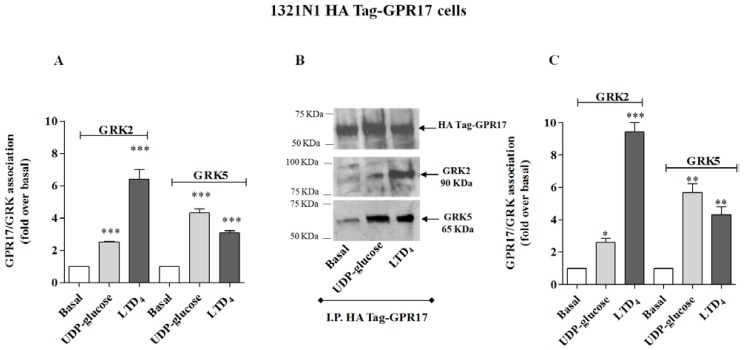
(**A**) 1321N1 HA Tag-GPR17 were treated for 5 min with medium alone (basal), or with 100 μM UDP-glucose, or 100 nM LTD_4_. Cell lysates (10 μg) were captured on wells pre-coated with anti-HA Tag antibody. After extensive washes, levels of GPR17/GRK complexes were quantified using specific anti-GRK2 or anti-GRK5 antibodies, and subsequently an HRP-conjugated antibody/TMB substrate kit. Blank wells were obtained in the absence of the primary anti-GRK antibodies. Data are expressed as fold *vs.* basal, and represent the mean ± SEM of at least three independent experiments; (**B**,**C**) 1321N1 HA Tag-GPR17 cells were treated as in (**A**). GPR17 was immunoprecipitated from the obtained cell lysates using an anti HA-Tag antibody, and immunoprecipitates were probed with anti-HA Tag, anti-GRK2 or anti-GRK5 antibodies; (**B**) representative immunoblots; (**C**) signals were quantified by densitometry and expressed as fold *vs.* basal value. Data are the means ± SEM (*N* = 3). Statistical significance was determined with a one-way ANOVA with Bonferroni post-test: * *p* < 0.05, ** *p* < 0.01, *** *p* < 0.001 *vs.* basal value.

**Figure 3.**
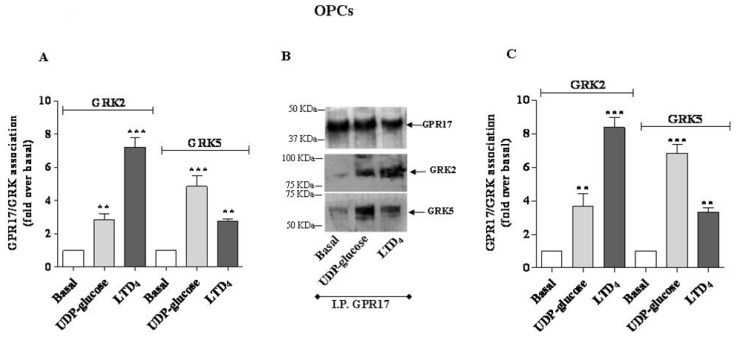
(**A**) Primary OPCs were treated for 5 min with medium alone (basal), or with 5 μM UDP-glucose, or 50 nM LTD_4_. Cell lysates (10 μg) were captured on wells pre-coated with anti-GPR17 antibody. After extensive washes, levels of GPR17/GRK complexes were quantified using specific anti-GRK2 or anti-GRK5 antibodies, and subsequently an HRP-conjugated antibody/TMB substrate kit. Blank wells were obtained in the absence of the primary anti-GRK antibodies. Data are expressed as fold *vs.* basal, and represent the mean ± SEM of at least three independent experiments; (**B**,**C**) Primary OPCs were treated as in (**A**). GPR17 was immunoprecipitated from the obtained cell lysates using an anti-GPR17 antibody, and immunoprecipitates were probed with anti-GPR17, anti-GRK2 or anti-GRK5 antibodies; (**B**) representative immunoblots; (**C**) signals were quantified by densitometry and expressed as fold *vs.* basal value. Data are the means ± SEM (*N* = 3). Statistical significance was determined with a one-way ANOVA with Bonferroni post-test: ** *p* < 0.01, *** *p* < 0.001 *vs.* basal value.

The immunoenzymatic assay, performed using primary anti-GRK2 or anti-GRK5 antibodies, revealed a significant enhancement of specific absorbance in agonist-stimulated samples of 1321N1 HA Tag GPR17 cells with respect to untreated cells (Figure 2A), demonstrating that GPR17 activation was able to induce receptor association with GRK2 and GRK5. In particular, UDP-glucose induced a preferential receptor association with GRK5, while LTD_4_ caused a greater association to GRK2. Comparable results were obtained with OPC lysates (Figure 3A).

To validate the quantitative results of the ELISA assay, a co-immunoprecipitation/western blot analysis was performed. 1321N1 HA Tag-GPR17 cells or OPCs were treated with medium alone, or with UDP-glucose or LTD_4_, collected and then lysed by the addition of RIPA. GPR17 was immunoprecipitated from cell lysates obtained from transfected HA Tag-GPR17 cells or OPCs, using an anti-HA Tag antibody or with the anti-GPR17 antibody, respectively. Immunocomplexes were then resolved by SDS-PAGE, and probed overnight at 4 °C with the specific primary anti-GRK2 or anti-GRK5 antibodies. Results show that, both in transfected cells (Figure 2B) and in primary OPCs (Figure 3B), GPR17 weakly associated with GRK2 or GRK5 under basal conditions, and this association was significantly enhanced after 5 min receptor stimulation with UDP-glucose or LTD_4_ (Figures 2B and 3B). According to the data obtained in the ELISA assay, densitometric analysis of immunoreactive bands revealed that both in transfected cells (Figure 2C) and in primary OPCs (Figure 3C), LTD_4_ preferentially induced the association between GPR17 and the GRK2 isoform with respect to GRK5. *Vice versa*, UDP-glucose mainly favoured the association between the receptor and GRK5, compared to GRK2 [19].

### 2.3. Use of the ELISA Assay in the Study of GPR17 Association with β-Arrestins

GRK-phosphorylated GPCRs generally bind to β-arrestins, which desensitize receptor-mediated G protein signalling via several mechanisms, including direct steric hindrance of the receptor by β-arrestin attachment, recruitment of second messenger-degrading enzymes, and by acting as a scaffold for proteins facilitating receptor internalization [20]. Upon β-arrestin translocation to the plasma membrane, it has been hypothesized that the stability of the receptor/β-arrestin complex represents a significant factor in determining the activation of distinct intracellular pathways [21].

On this basis, we then investigated GPR17/β-arrestin complexes after agonist-mediated receptor stimulation. To this purpose, 1321N1 HA Tag-GPR17 cells or OPCs were incubated for 5 or 30 min with UDP-glucose or LTD_4_, and the association between the receptor and β-arrestins was evaluated by the ELISA assay.

Cell lysates were incubated on pre-coated wells, and the presence of GPR17/β-arrestin complexes was detected using specific primary antibodies against β-arrestins, and evaluating the colorimetric quantification after the addition of the appropriate HRP-conjugate secondary antibody/TMB substrate.

**Figure 4.**
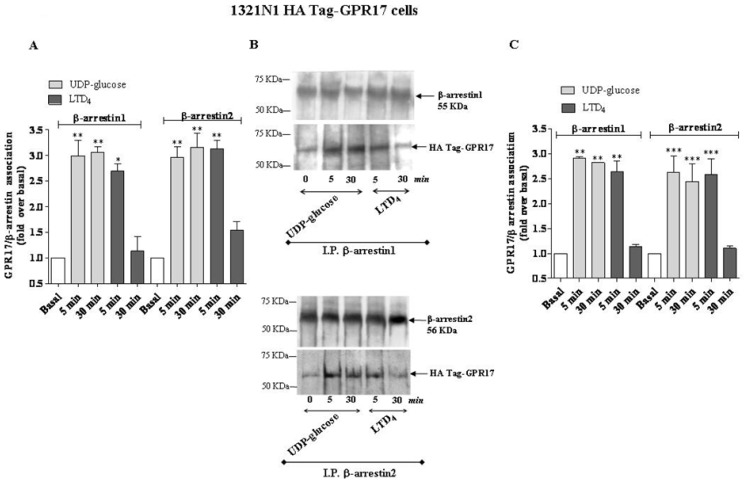
(**A**) 1321N1 HA Tag-GPR17 were treated for 5 or 30 min with medium alone (basal), or with 100 μM UDP-glucose, or 100 nM LTD_4_. Cell lysates (10 μg) were captured on wells pre-coated with anti-HA Tag antibody. After extensive washes, levels of GPR17/β-arrestin complexes were quantified using specific anti-β-arrestin1 or β-arrestin1-2 antibodies, and subsequently an HRP-conjugated antibody/TMB substrate kit. Blank wells were obtained in the absence of the primary anti-β-arrestin antibodies. Data are expressed as fold *vs.* basal, and represent the mean ± SEM of at least three independent experiments; (**B**,**C**) 1321N1 HA Tag-GPR17 cells were treated as in (**A**). GPR17 was immunoprecipitated from the obtained cell lysates using an anti-HA Tag antibody, and immunoprecipitates were probed with anti-β-arrestin1 or anti-β-arrestin2 antibodies; (**B**) representative immunoblots; (**C**) signals were quantified by densitometry and expressed as fold *vs.* basal value. Data are the means ± SEM (*N* = 3). Statistical significance was determined with a one-way ANOVA with Bonferroni post-test: * *p* < 0.05, ** *p* <0.01, *** *p* < 0.001 *vs.* basal value.

**Figure 5.**
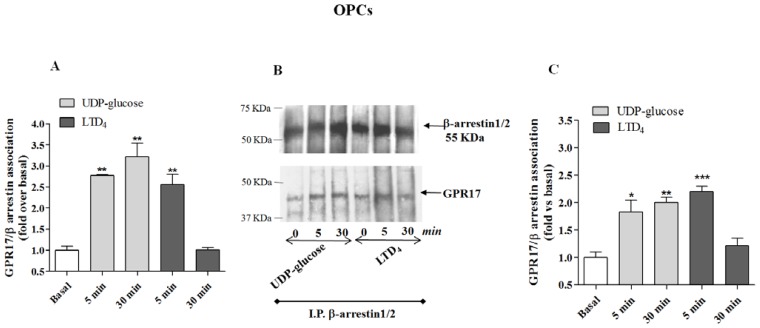
(**A**) Primary OPCs were treated for 5 or 30 min with medium alone (basal), or with 5 μM UDP-glucose, or 50 nM LTD_4_. Cell lysates (10 μg) were captured on wells pre-coated with anti-GPR17 antibody. After extensive washes, levels of GPR17/β-arrestin complexes were quantified using a specific anti-β-arrestin1/2 antibody, and subsequently an HRP-conjugated antibody/TMB substrate kit. Blank wells were obtained in the absence of the primary anti-β-arrestin antibodies. Data are expressed as fold *vs.* basal value, and represent the mean ± SEM of at least three independent experiments; (**B**,**C**) Primary OPCs were treated as in (**A**). GPR17 was immunoprecipitated from the obtained cell lysates using an anti-GPR17 antibody, and immunoprecipitates were probed with anti-β-arrestin1/2 antibody. (**B**) representative immunoblots; (**C**) signals were quantified by densitometry and expressed as fold *vs.* basal value. Data are the means ± SEM (*N* = 3). Statistical significance was determined with a one-way ANOVA with Bonferroni post-test: * *p* < 0.05, ** *p* < 0.01, *** *p* < 0.001 *vs.* basal value.

The immunoenzymatic assay revealed a significant enhancement of specific absorbance in the 5 min agonist-stimulated samples of 1321N1 HA Tag-GPR17 cells with respect to untreated cells (Figure 4A), demonstrating that GPR17 activation for 5 min with its ligands was able to induce receptor association with β-arrestins. The enhancement of specific absorbance was still detectable after 30 min of incubation with UDP-glucose, suggesting that this ligand induces the formation of stable GPR17-β-arrestin complexes; on the contrary, upon stimulation with LTD_4_, GPR17 formed a transient complex with arrestins that dissociated within 30 min (Figure 4A). Comparable results were obtained with OPC lysates (Figure 5A), demonstrating that also in primary cells only UDP-glucose can induce a stable association with β-arrestins.

To validate the quantitative results of the ELISA assay, a co-immunoprecipitation and western blot analysis was performed. β-arrestins were immunoprecipitated using specific antibodies from cell lysates obtained from transfected HA Tag-GPR17 cells or OPCs. Immunocomplexes were then resolved by SDS-PAGE, and probed with the specific primary anti-HA Tag or anti-GPR17 antibodies. The results show that, both in transfected cells (Figure 4B) and in primary OPCs (Figure 5B), GPR17 weakly associated with β-arrestins under basal conditions, while this association was significantly enhanced after 5 min receptor stimulation with UDP-glucose or LTD_4_ (Figures 4B and 5B). According to the data obtained in the ELISA assay, densitometric analysis of immunoreactive bands revealed that both in transfected cells (Figure 4C) and in primary OPCs (Figure 5C), the immunocomplexes were still detectable after 30 min of incubation with UDP-glucose, but not with LTD_4_ [19].

## 3. Experimental Section

### 3.1. Cell Culture

Astrocytoma cells (1321N1), transfected with HA Tag-GPR17 were maintained in DMEM-F12 containing 10% FBS, penicillin (200 U/mL), streptomycin (200 μg/mL), and l-glutamine (2 mM) at 37 °C, 5% CO_2_ and 95% humidity, with 300 μg/mL G418.

### 3.2. Primary OPC Cultures

OPCs were isolated from mixed glial cultures from postnatal day 2 Sprague-Dawley rat cortex, by the shaking method, as described [13]. OPCs were cultured in Neurobasal with 2% B27 (Invitrogen, Monza, Italy), 2 mM l-glutamine, 10 ng/mL human platelet-derived growth factor BB (Sigma-Aldrich, Milan, Italy), and 10 ng/mL human basic fibroblast growth factor (Invitrogen, Monza, Italy) to promote proliferation. About the 90% of cells was positive for the typical OPC marker Olig2; a very low percentage of contaminating astrocytes and microglia was found. After one day, cells were switched to a Neurobasal medium lacking growth factors to allow differentiation. Experiments were performed after Five to six days of differentiation in culture, when, in our standardized protocol [13], cells reached the immature pre-oligodendrocyte stage; indeed, previous studies [13] have shown that GPR17 expression is maximal in immature pre-oligodendrocytes and then gradually decreases along with terminal maturation.

### 3.3. Cell Treatment and Preparation of Cell Lysates

1321N1 HA Tag-GPR17 cells or OPCs were treated with medium alone (basal), or UDP-glucose (100 or 5 μM, respectively) or with LTD_4_ (100 or 50 nM, respectively) for 5 or 30 min. At the end of treatments, cells were washed twice in ice-cold phosphate-buffered saline, collected by centrifugation, and resuspended in Lysis buffer (20 mM Tris HCl, 137 mM NaCl, 10% glycerol, 1% NONIDET40, 2 mM EDTA, pH 8), containing 1% of the Protease inhibitor Cocktail (Sigma Aldrich, Milan, Italy). Optimal composition of lysis buffer was determined in preliminary experiments.

### 3.4. General ELISA Assay

Wells were pre-coated overnight at room temperature with 60 μL of anti-HA Tag antibody (#05-904, Millipore, 1:500 in 0.05% Poly-l-Ornithine) or of anti-*N* terminus GPR17 antibody (sc-74792, Santa Cruz Biotechnology, 1:100 in 0.05% Poly-l-Ornithine), in the case of transfected cells or OPCs, respectively. After three washes of 5 min with 100 μL of PBS/Tween 0.1%, cell lysates (10 μg in a final volume of 100 μL) were transferred to the pre-coated wells for 120 min with a plastic cover or film and kept under constant agitation.

Three quick washes were performed with PBS/Tween 0.1% to remove unbound HA Tag or GPR17, and each well was incubated for 15 min with bovine serum albumin 1% (BSA, Sigma Aldrich, Milan, Italy) to block non specific sites. Then, wells were incubated for 120 min at room temperature with the specific primary antibodies: anti-GRK2 (Santa Cruz Biotechnology, Dallas, TX, USA, sc-562; 1:400); anti-GRK5 (Santa Cruz Biotechnology, Dallas, TX, USA, sc-565; 1:400); anti-GPR17 (1:1000); anti-β-arrestin1 (Santa Cruz Biotechnology, Dallas, TX, USA, sc-9182; 1:400); or anti-β-arrestin2 (ABCAM, ab31294; 1:500); anti-β-arrestin1/2 (Santa Cruz Biotechnology, Dallas, TX, USA, sc-74591; 1:400).

Wells were washed and incubated for 1 h with a secondary HRP-conjugate antibody (diluted in 5% milk), and washed again. The TMB substrate kit (Thermo Fisher Scientific Inc., Waltham, MA, USA) allowed a colorimetric quantification of the complex of GPR17 with GRKs or β-arrestins.

Blanks were obtained processing cell lysates in the absence of the primary antibodies. To avoid cross interactions between the different antibodies, the chosen primary antibody was always from a different species with respect to the antibody used for well pre-coating.

Absorbance’s values at 450 nm were measured, background subtracted and bar graphs were generated using Graph Pad Prism 4 software (GraphPad Software Inc., San Diego, CA, USA), from which the percentage of association with GRK and β-arrestins, compared to basal value, were derived.

### 3.5. Co-Immunoprecipitation Assay

1321N1 HA Tag-GPR17 cells and OPCs were treated with medium alone (basal) or with UDP-glucose (100 or 5 μM, respectively) or LTD_4_ (100 or 50 nM, respectively) for 5 or 30 min, and then lysed for 60 min at 4 °C by the addition of RIPA buffer (9.1 mM NaH_2_PO_4_, 1.7 mM Na_2_HPO_4_, 150 mM NaCl, pH 7.4, 0.5% sodium deoxycholate, 1% Nonidet P-40, and 0.1% SDS, protease inhibitor cocktail). Extracts were then equalised by protein assay. In the case of GRKs, one mg cell lysates were incubated with anti-HA Tag antibody (for transfected cells) or with a home-made rabbit anti-GPR17 antibody (for OPCs) overnight at 4 °C under constant rotation. In the case of β-arrestins, 1.5 mg cell lysates from transfected cells were incubated with anti-β-arrestin1 (Santa Cruz Biotechnology, Dallas, TX, USA, sc-9182; 5 μg/sample), or anti-β-arrestin2 (abcam, ab31294; 5 μg/sample), or anti-β-arrestin1/2 (Santa Cruz Biotechnology, Dallas, TX, USA, sc-74591; 5 μg/sample), overnight at 4 °C. Probes were then immunoprecipitated with protein A-Sepharose (2–3 h at 4 °C). Immunocomplexes, after being washed, were resuspended in Laemmli solution and boiled for 5 min, resolved by SDS-PAGE (8.5%), transferred to PVDF membranes and probed overnight at 4 °C with the specific primary antibodies: anti-GRK2 (Santa Cruz Biotechnology, Dallas, TX, USA, sc-562; 1:200); anti-GRK5 (Santa Cruz Biotechnology, Dallas, TX, USA, sc-565; 1:100); anti-HA Tag (1:500); anti-GPR17 (1:1000). The primary antibodies were detected using anti-rabbit or anti-mouse IgG light chains conjugated to peroxidase (diluted 1:10.000 and 1:5.000, respectively).

### 3.6. Data Analysis

Statistical analysis of data was performed using a one-way ANOVA study followed by the Bonferroni test for repeated measurements. Differences were considered statistically significant with *p* < 0.05. The densitometric analysis of immunoreactive bands was performed using the ImageJ program (ImageJ, US National Institutes of Health, Bethesda, MA, USA, 1997).

## 4. Conclusions

Herein, using GPR17 as a prototype, an immunoenzymatic assay to detect GPCR interaction with its signaling proteins was described.

GPCRs can associate with a wide range of either soluble or transmembrane interacting proteins, that are implicated in receptor proper folding, targeting to the appropriate subcellular compartments and in fulfilling their signaling tasks. Detecting protein-protein interactions and multi-protein complexes formation are thus crucial to understand protein function and cell biology.

Although several methods have been already described to detect protein complexes, some of them are quite laborious, expensive, and do not generate fully quantitative results. Moreover, only a small number of the interactions are supported by more than one method.

A simple sandwich immunoenzymatic assay was developed and optimized on cell lysates obtained from HA Tag-GPR17 transfected cells and from primary OPC cultures, which natively express GPR17 at high levels. This method allows to use small amounts of cell lysates and of antibodies, and to save handling time, as it allows to obtain quantitative results in half a day. Importantly, the quantitative information on GPR17 interaction with its regulatory proteins were supported by data obtained in parallel by co-immunoprecipitation experiments. This ELISA assay can be applied to every GPCR, in the study of the interaction with signaling proteins, as well as with another GPCR (eterodimers).
